# Hematological factors in women with Asherman syndrome and primary infertility: An overview

**DOI:** 10.1097/MD.0000000000045562

**Published:** 2025-10-24

**Authors:** Emmanuel Ifeanyi Obeagu, Getrude Uzoma Obeagu

**Affiliations:** a Department of Biomedical and Laboratory Science, Africa University, Mutare, Zimbabwe; b School of Nursing Science, Kampala International University, Ishaka, Uganda.

**Keywords:** angiogenic factors, Asherman syndrome, coagulation abnormalities, hematological factors, inflammatory markers, intrauterine adhesions, primary infertility

## Abstract

Asherman syndrome (AS) presents a complex challenge in the realm of female infertility, characterized by intrauterine adhesions that result from uterine trauma. While the primary etiology of this condition is well-established, recent investigations have begun to uncover potential associations between hematological factors and the development or exacerbation of AS. This review offers a comprehensive overview of hematological factors in women with AS and primary infertility. The paper delves into the intricate interplay of coagulation abnormalities, inflammatory markers, and angiogenic factors in the pathogenesis of this condition. Furthermore, we discuss the clinical implications of hematological abnormalities on fertility outcomes and explore potential therapeutic strategies for managing AS in affected women. By synthesizing existing literature and highlighting emerging research trends, this review aims to provide insights that may inform future research directions and clinical practice in the management of AS and associated infertility.

## 1. Introduction

Asherman syndrome (AS), a gynecological disorder characterized by the formation of intrauterine adhesions (IUAs) or fibrosis, represents a significant impediment to fertility in women.^[1]^ First described in 1948 by Joseph G. Asherman, this condition typically arises as a consequence of uterine trauma, often following surgical procedures such as dilation and curettage (D&C) or cesarean section.^[2]^ The resultant adhesions within the uterine cavity can lead to menstrual disturbances, recurrent pregnancy loss, and primary infertility, posing considerable challenges to affected individuals seeking to conceive.^[3]^ While the primary etiology of AS involves uterine trauma and subsequent disruption of the endometrial basal layer, recent research endeavors have begun to explore the potential role of hematological factors in the pathogenesis of this condition.^[4]^ Hematological abnormalities, encompassing disturbances in coagulation, inflammation, and angiogenesis, may contribute to the formation and persistence of IUAs, thus influencing fertility outcomes in affected women.^[5]^ Understanding the intricate interplay between hematological factors and AS holds promise for elucidating novel mechanisms underlying this condition and informing targeted therapeutic interventions.

Coagulation abnormalities represent a prominent area of interest in the context of AS, with emerging evidence suggesting dysregulated fibrinolysis and impaired endometrial vascularization following uterine trauma.^[6]^ Altered clotting factor profiles, characterized by elevated tissue factor levels and diminished antithrombin III activity, may predispose individuals to excessive fibrin deposition and subsequent adhesion formation within the uterine cavity.^[7]^ These findings underscore the potential significance of coagulation dysfunction in the pathogenesis of AS and its implications for fertility. In addition to coagulation abnormalities, inflammatory processes have garnered attention as potential contributors to the development and persistence of IUAs in AS.^[8]^ Chronic inflammation, characterized by elevated levels of pro-inflammatory cytokines such as interleukin-6 (IL-6) and tumor necrosis factor-alpha (TNF-α), may promote fibrotic changes and impair endometrial regeneration following uterine trauma.^[9]^ The dysregulated inflammatory milieu within the uterine cavity may exacerbate tissue damage and hinder the resolution of adhesions, thereby exacerbating infertility in affected women.^[10]^ Furthermore, dysregulation of angiogenic factors has emerged as a key area of investigation in the pathogenesis of AS.^[11]^ Angiogenesis, the process of new blood vessel formation, plays a crucial role in endometrial repair and regeneration following uterine trauma.^[12]^ Perturbations in angiogenic signaling pathways, including vascular endothelial growth factor (VEGF) and angiopoietin-2 (Ang-2), may compromise endometrial vascularization and impede tissue healing, thus contributing to the formation and persistence of IUAs in AS.

## 2. Aim

The aim of this study is to provide a comprehensive overview of hematological factors in women diagnosed with AS and primary infertility. By synthesizing existing literature, the study seeks to elucidate the prevalence, significance, and potential mechanisms through which hematological abnormalities impact reproductive outcomes in this patient population. Additionally, the paper aims to explore the diagnostic challenges associated with identifying hematological factors in women with AS and primary infertility, as well as the therapeutic implications of addressing these factors in clinical practice. Ultimately, the review aims to contribute to a better understanding of the interplay between hematological factors and AS-associated infertility, informing evidence-based management strategies and optimizing reproductive outcomes for affected women.

## 3. Rationale

The rationale for this study stems from the recognition of AS as a complex and multifactorial condition that poses significant challenges to women desiring fertility. While IUAs are the hallmark of AS, recent evidence suggests a potential association with hematological abnormalities. Understanding the role of hematological factors in the pathogenesis and management of AS is essential for improving reproductive outcomes in affected women. Firstly, investigating the prevalence and significance of hematological abnormalities in women with AS is crucial for identifying additional factors contributing to infertility in this population. By elucidating the potential mechanisms through which hematological factors contribute to IUAs and impaired fertility, this study aims to provide insights into the underlying pathophysiology of AS. Secondly, addressing the diagnostic challenges associated with identifying hematological factors in women with AS and primary infertility is essential for effective patient management. Integrating hematological assessment into the diagnostic workup of these patients may facilitate early detection and personalized treatment strategies, thereby optimizing reproductive outcomes. Furthermore, exploring the therapeutic implications of addressing hematological factors in the management of AS-associated infertility has significant clinical relevance. By evaluating the efficacy of interventions such as anticoagulant therapy, immunomodulatory agents, and assisted reproductive technologies, this study aims to inform evidence-based treatment approaches tailored to the individual needs of affected women.

## 4. Review methodology

The review methodology for this study involved a systematic approach to identify relevant literature addressing hematological factors in women with AS and primary infertility. The following steps were undertaken:

### 4.1. Literature search

A comprehensive search strategy was developed to identify relevant studies. Electronic databases such as PubMed, MEDLINE, Embase, and Google Scholar were searched using keywords and Medical Subject Headings (MeSH) terms related to AS, primary infertility, and hematological factors. The search was conducted with no restriction on publication date, but English language articles were prioritized.

### 4.2. Inclusion and exclusion criteria

Studies were included if they investigated hematological factors in women diagnosed with AS and primary infertility. Both observational studies (e.g., cohort studies, case-control studies) and interventional studies (e.g., clinical trials, case series) were considered. Studies focusing on other uterine pathologies or unrelated to hematological factors were excluded.

### 4.3. Study selection

Two independent reviewers screened the titles and abstracts of identified articles to assess their relevance to the research question. Full-text articles of potentially relevant studies were then retrieved and assessed for eligibility based on the inclusion and exclusion criteria.

### 4.4. Data extraction

Data from selected studies were extracted using a standardized form. Key information extracted included study design, participant characteristics, hematological parameters investigated, diagnostic methods used, and findings related to hematological factors in AS-associated infertility.

### 4.5. Quality assessment

The quality of included studies was assessed using appropriate tools depending on the study design (e.g., Newcastle–Ottawa scale for cohort studies, Cochrane risk-of-bias tool for clinical trials). Studies with high methodological quality were given more weight in the synthesis of findings.

### 4.6. Data synthesis

A narrative synthesis approach was employed to summarize and integrate findings from the included studies. Themes related to the prevalence, significance, mechanisms, diagnostic challenges, and therapeutic implications of hematological factors in AS-associated infertility were identified and synthesized.

### 4.7. Critical appraisal

The strengths and limitations of the included studies were critically appraised, and gaps in the existing literature were identified. Recommendations for future research directions were formulated based on the synthesis of findings and identified gaps.

## 5. Pathogenesis of Asherman syndrome

The pathogenesis of AS involves a cascade of events triggered by uterine trauma, leading to the formation of IUAs or fibrosis.^[2]^ The condition typically arises following procedures such as dilation and curettage (D&C), cesarean section, or myomectomy, which result in injury to the endometrium and underlying basal layer of the uterine wall. The disruption of the endometrial basal layer initiates a process of aberrant wound healing, characterized by the formation of scar tissue and adhesions within the uterine cavity.^[13]^ Uterine trauma induces an inflammatory response, marked by the recruitment of immune cells and the release of pro-inflammatory cytokines such as interleukin-6 (IL-6) and tumor necrosis factor-alpha (TNF-α).^[14]^ This inflammatory milieu promotes tissue remodeling and repair; however, in the context of AS, dysregulated inflammation may lead to excessive fibrotic changes and impaired regeneration of the endometrium.^[15]^ Prolonged or unresolved inflammation perpetuates tissue damage and contributes to the formation of adhesions.

The healing process in AS is characterized by the deposition of fibrin-rich granulation tissue, which serves as a scaffold for scar formation.^[16]^ Dysregulated fibrinolysis, involving an imbalance between fibrin deposition and degradation, may result in the accumulation of fibrin within the uterine cavity.^[17]^ Excessive fibrin deposition contributes to the formation of dense adhesions, which can obliterate the endometrial cavity and compromise fertility by impairing menstrual flow and preventing embryo implantation.^[18]^ Coagulation abnormalities have been implicated in the pathogenesis of AS, with alterations in clotting factors and fibrinolytic pathways observed in affected individuals.^[19]^ Dysregulated coagulation may predispose women to thrombotic events within the uterine vasculature, leading to ischemic injury and impaired tissue repair.^[20]^ Furthermore, impaired endometrial vascularization following uterine trauma may exacerbate coagulation dysfunction, creating a vicious cycle of tissue damage and adhesion formation. Angiogenesis, the process of new blood vessel formation, is critical for tissue repair and regeneration. Dysregulation of angiogenic factors, including VEGF and angiopoietin-2 (Ang-2), has been implicated in the pathogenesis of AS.^[21]^ Impaired angiogenic signaling compromises endometrial vascularization and hinders tissue healing, promoting the persistence of IUAs.^[22]^ Collectively, dysregulated inflammation, aberrant coagulation, and impaired angiogenesis contribute to the pathogenesis of AS, culminating in the formation of IUAs and subsequent infertility (Table 1).

**Table 1:** Hematological factors and mechanistic roles in Asherman syndrome.

Hematological factor	Mechanistic role in Asherman syndrome
Inherited/Acquired thrombophilia	Promotes microthrombi formation, hypoxia, and fibrotic endometrial remodeling through TGF-β signaling.
Platelet activation	Excess release of TGF-β, VEGF, and PDGF enhances extracellular matrix deposition and adhesion formation.
Inflammatory blood markers (NLR, PLR, RDW)	Reflect chronic inflammation and oxidative stress that impair endometrial repair and receptivity.
Anemia (iron deficiency)	Decreases oxygen delivery, activates HIF pathways, and delays endometrial healing.
Endothelial dysfunction/Coagulation abnormalities	Triggers fibrin deposition and promotes persistent fibrotic tissue development.

HIF = hypoxia-inducible factor, NLR = neutrophil-to-lymphocyte ratio, PDGF = platelet-derived growth factor, PLR = platelet-to-lymphocyte ratio, RDW = red cell distribution width, TGF-β = transforming growth factor beta, VEGF = vascular endothelial growth factor.

## 6. Coagulation abnormalities

Coagulation abnormalities play a significant role in the pathogenesis of AS, contributing to the formation and persistence of IUAs.^[23]^ Following uterine trauma, dysregulated coagulation cascades may predispose individuals to excessive fibrin deposition within the uterine cavity, ultimately leading to the development of adhesions.^[24]^ One key aspect of coagulation abnormalities in AS involves disturbances in fibrinolysis, the process by which fibrin clots are broken down and removed from the body. Dysregulated fibrinolysis may result in the accumulation of fibrin within the uterine cavity, providing a substrate for the formation of dense adhesions. Imbalance in fibrinolytic pathways, characterized by elevated levels of fibrinogen and impaired activity of fibrinolytic enzymes such as plasmin, contributes to the persistence of fibrin-rich granulation tissue and impedes endometrial regeneration. Altered clotting factor profiles have also been observed in women with AS, further highlighting the role of coagulation abnormalities in this condition.^[25]^ Elevated levels of tissue factor, a key initiator of the extrinsic coagulation pathway, may promote thrombotic events within the uterine vasculature following uterine trauma.^[26]^ Conversely, decreased levels of antithrombin III, a natural anticoagulant, may exacerbate coagulation dysfunction and predispose individuals to hypercoagulability, increasing the risk of thrombus formation and ischemic injury within the endometrium. The dysregulated coagulation environment in AS not only promotes the formation of IUAs but also hinders endometrial repair and regeneration. Impaired endometrial vascularization, resulting from thrombotic occlusion of uterine blood vessels and decreased angiogenic signaling, compromises tissue healing and exacerbates the persistence of adhesions.^[27]^ Furthermore, ischemic injury to the endometrium perpetuates inflammation and fibrosis, creating a hostile microenvironment that impedes fertility and pregnancy establishment.

## 7. Inflammatory markers

Inflammatory markers play a crucial role in the pathogenesis of AS, influencing the inflammatory response following uterine trauma and contributing to the development and persistence of IUAs.^[28]^ Dysregulated inflammation within the uterine cavity promotes tissue damage, fibrosis, and impaired endometrial regeneration, ultimately compromising fertility in affected women.^[29]^ Chronic inflammation is a hallmark feature of AS, characterized by elevated levels of pro-inflammatory cytokines and chemokines within the endometrium.^[30]^ Interleukin-6 (IL-6) and tumor necrosis factor-alpha (TNF-α), 2 key mediators of the inflammatory cascade, have been implicated in the pathogenesis of IUAs.^[31]^ Elevated IL-6 levels are associated with increased fibrotic changes and impaired endometrial repair, while TNF-α promotes tissue damage and inhibits epithelial cell proliferation, exacerbating the formation of adhesions.

In addition to pro-inflammatory cytokines, alterations in inflammatory cell populations have been observed in women with AS.^[30]^ Increased infiltration of immune cells, such as macrophages and T lymphocytes, into the uterine cavity following uterine trauma perpetuates the inflammatory response and contributes to tissue fibrosis.^[31]^ Macrophages play a central role in tissue remodeling and repair; however, dysregulated macrophage activation may lead to excessive collagen deposition and adhesion formation within the endometrium.^[32]^ The dysregulated inflammatory milieu within the uterine cavity not only promotes the formation of IUAs but also hinders endometrial regeneration and repair.^[33]^ Impaired epithelial cell proliferation and migration, secondary to chronic inflammation, compromise the integrity of the endometrial basal layer and hinder the restoration of normal endometrial architecture.^[34]^ Furthermore, persistent inflammation perpetuates tissue fibrosis and scarring, creating a hostile microenvironment that impairs fertility and pregnancy establishment.

## 8. Angiogenic factors

Angiogenic factors play a critical role in the pathogenesis of AS, influencing the process of endometrial repair and regeneration following uterine trauma.^[35]^ Dysregulation of angiogenic signaling pathways contributes to impaired endometrial vascularization, compromised tissue healing, and the formation of IUAs, ultimately impacting fertility outcomes in affected women. VEGF, a key angiogenic factor, promotes the growth and proliferation of endothelial cells, facilitating the formation of new blood vessels within the endometrium.^[36]^ Following uterine trauma, dysregulated VEGF signaling may impair endometrial vascularization, leading to ischemic injury and compromised tissue repair. Reduced VEGF expression or activity within the uterine cavity hinders angiogenesis and impedes the restoration of normal endometrial architecture, exacerbating the formation of adhesions.

Angiopoietins, including angiopoietin-1 (Ang-1) and angiopoietin-2 (Ang-2), are essential regulators of angiogenesis and vascular stability.^[37]^ Ang-1 promotes endothelial cell survival and vascular maturation, while Ang-2 acts as a context-dependent agonist or antagonist of angiogenesis.^[38]^ Dysregulated Ang-2 expression, characterized by increased levels or altered signaling, may disrupt vascular remodeling and contribute to the persistence of immature and leaky blood vessels within the endometrium, exacerbating tissue damage and adhesion formation. The balance between pro-angiogenic and anti-angiogenic factors within the uterine cavity is critical for normal endometrial repair and regeneration. Dysregulation of this balance, characterized by aberrant expression or signaling of angiogenic factors, promotes a hostile microenvironment that impedes tissue healing and exacerbates the formation of IUAs.^[39]^ Furthermore, compromised endometrial vascularization compromises fertility by impairing menstrual flow, hindering embryo implantation, and increasing the risk of recurrent pregnancy loss.

## 9. Clinical implications

The understanding of hematological factors in AS holds significant clinical implications for the diagnosis, management, and prognosis of affected individuals.^[40]^ Incorporating hematological assessment into the evaluation of women with suspected or confirmed AS may enhance diagnostic accuracy and inform personalized treatment strategies tailored to individual patient needs. Hematological evaluation, including assessment of coagulation parameters, inflammatory markers, and angiogenic factors, may serve as valuable diagnostic tools for identifying individuals at risk of developing AS or those with existing IUAs.^[41]^ Comprehensive hematological profiling may facilitate early detection of coagulation abnormalities, chronic inflammation, or dysregulated angiogenic signaling, enabling timely intervention and preventing the progression of IUAs. Hematological factors may also offer prognostic insights into fertility outcomes and pregnancy complications in women with AS.^[41]^ Abnormalities in coagulation parameters, such as hypercoagulability or impaired fibrinolysis, may increase the risk of thrombotic events and adverse pregnancy outcomes, including recurrent pregnancy loss or placental dysfunction.^[31]^ Furthermore, dysregulated inflammatory markers or angiogenic factors may impact endometrial receptivity and embryo implantation, influencing the success of assisted reproductive technologies and natural conception.

Targeted modulation of hematological factors may represent novel therapeutic avenues for the management of AS and associated infertility.^[42]^ Anticoagulant therapy, such as low-molecular-weight heparin or aspirin, may mitigate hypercoagulability and prevent thrombus formation within the uterine vasculature, promoting endometrial vascularization and tissue healing. Anti-inflammatory agents, including corticosteroids or nonsteroidal anti-inflammatory drugs (NSAIDs), may attenuate chronic inflammation and reduce fibrotic changes within the endometrium, facilitating endometrial repair and regeneration. Additionally, administration of exogenous angiogenic factors, such as VEGF or angiopoietin-1 (Ang-1), may enhance endometrial vascularization and mitigate the formation of IUAs, thereby improving fertility outcomes in affected individuals. Regular monitoring of hematological parameters may be warranted in women undergoing treatment for AS to assess treatment response and disease progression.^[41]^ Serial evaluation of coagulation parameters, inflammatory markers, and angiogenic factors may guide therapeutic adjustments and inform clinical decision-making. Long-term follow-up of affected individuals is essential to monitor fertility outcomes, assess the recurrence of IUAs, and address potential complications, such as recurrent pregnancy loss or obstetric complications, thereby optimizing reproductive health and pregnancy outcomes in this population (Table 2).

**Table 2:** Diagnostic and therapeutic implications of hematological evaluation in Asherman syndrome.

Clinical application	Key details
Baseline hematological evaluation	CBC, coagulation profile, and inflammatory indices (NLR, PLR, RDW) to detect underlying hematologic abnormalities.
Thrombophilia screening	Identify factor V Leiden, prothrombin mutations, protein C/S deficiency, antiphospholipid antibodies for risk stratification.
Anticoagulation/Antiplatelet therapy	Low-dose aspirin or LMWH in selected patients undergoing hysteroscopic adhesiolysis or IVF to reduce adhesion recurrence.
Platelet-rich plasma (PRP) therapy	Uses autologous platelets to deliver growth factors for endometrial regeneration and improved fertility outcomes.
Inflammation modulation	Anti-inflammatory agents or antioxidants to reduce systemic hematologic disturbances contributing to fibrosis.

CBC = complete blood count, HIF = hypoxia-inducible factor, LMWH = low-molecular-weight heparin NLR = neutrophil-to-lymphocyte ratio, PDGF = platelet-derived growth factor, PLR = platelet-to-lymphocyte ratio, RDW = red cell distribution width, TGF-β = transforming growth factor beta, VEGF = vascular endothelial growth factor.

## 10. Conclusion

AS presents a multifaceted challenge in the realm of female infertility, characterized by the formation of IUAs following uterine trauma. While the primary etiology of this condition is well-established, emerging research suggests that hematological factors play a significant role in its pathogenesis and clinical manifestations. Coagulation abnormalities, inflammatory responses, and dysregulation of angiogenic factors contribute to the formation and persistence of IUAs, compromising fertility outcomes in affected women.

## Author contributions

**Conceptualization:** Emmanuel Ifeanyi Obeagu.

**Methodology:** Emmanuel Ifeanyi Obeagu, Getrude Uzoma Obeagu.

**Resources:** Emmanuel Ifeanyi Obeagu.

**Supervision:** Emmanuel Ifeanyi Obeagu.

**Validation:** Emmanuel Ifeanyi Obeagu.

**Visualization:** Emmanuel Ifeanyi Obeagu.

**Writing – original draft:** Emmanuel Ifeanyi Obeagu, Getrude Uzoma Obeagu.

**Writing – review & editing:** Emmanuel Ifeanyi Obeagu, Getrude Uzoma Obeagu.
